# Prevalence and factors associated with low back pain among nurses at a regional hospital in KwaZulu-Natal, South Africa

**DOI:** 10.4102/hsag.v23i0.1082

**Published:** 2018-05-29

**Authors:** Thembelihle Dlungwane, Anna Voce, Stephen Knight

**Affiliations:** 1 Discipline of Public Health Medicine, University of KwaZulu-Natal, South Africa

## Abstract

**Background:**

Low back pain (LBP) is a public health problem worldwide and is a common cause of work-related disorder among workers, especially in the nursing profession. Recruitment and retention of nurses is a challenge, and the nursing shortage has been exacerbated by the burden of occupational injuries such as LBP and related disabilities. The physiotherapy clinical records revealed that caseload of nurses presenting for the management of LBP was increasing. The prevalence and factors associated with LBP were unclear.

**Methods:**

A cross-sectional study design with an analytic component was implemented. Data were collected utilising a self-administered questionnaire to determine the prevalence and factors associated with LBP among nurses at a regional hospital. Bivariate analyses were performed to determine the factors associated with LBP.

**Results:**

The point prevalence of current LBP in nurses was 59%. The highest prevalence was recorded among enrolled nurses (54%), respondents aged 30–39 (46%), overweight respondents (58%) and those working in obstetrics and gynaecology (49%). Bending (*p* = 0.002), prolonged position (*p* = 0.03) and transferring patients (*p* = 0.004) were strongly associated with LBP. Nurses with more than 20 years in the profession reported a high prevalence of LBP. The prevalence of LBP was higher among the participants who were on six-month rotations (76%) compared with those on yearly rotation (16%).

**Conclusion:**

A high proportion of nurses reported to have LBP. Occupational factors are strongly associated with LBP. Education programmes on prevention and workplace interventions are required in order to reduce occupational injuries.

## Introduction

Low back pain (LBP) is a public health problem worldwide and is a common cause of work-related disorder among health care workers, especially in the nursing profession (Cunningham, Flynn & Blake 2006; Homaid et al. 2016; Olivier et al. 2009; Tezel 2005). Various studies have shown that the main occupational risk factors associated with LBP in nurses are lifting and moving patients, sustained postures, job organisation, poor ergonomic structures, improper work design, low social support, poor job satisfaction, staff shortages and poor working conditions (Al Dajah & Al Daghdi 2013; Ghilan et al. 2013). Consequently, LBP is associated with absenteeism and decreased productivity (Mannion et al. 2009).

The mean point prevalence of LBP in Africa among the adult population is 32% and the chronic LBP prevalence among Africans ranges from 14% to 72% (Louw, Morris & Grimmer-Somers 2007). Low back pain is common among health workers and the nurses are the majority hospital workforce (Johnson & Emmanuel 2016; Olivier et al. 2009). The prevalence of LBP reported in different studies among nurses in Africa ranges from 33% to 73.5% (Johnson & Emmanuel 2016; Munabi et al. 2014; Sikiru & Hanifa 2010; Sikiru & Shmaila 2009; Tinubu et al. 2010). The LBP point prevalence among employees in a district hospital in South Africa was found to be 47% (Olivier et al. 2009). District hospitals provide Level 1 hospital service that cannot be delivered at a clinic or a health centre, whereas regional hospitals offer Level 2 services that provide care requiring the intervention of specialists and general practitioners. However, regional hospitals are often the most overburdened of all levels of hospitals, bearing the brunt of the shortage of staff and equipment in district hospitals (Cullinan 2006).

Low back pain is an occupational hazard that affects the productivity of nurses (El-Soud et al. 2014). Recruitment and retention of nurses is a challenge, and the nursing shortage has been exacerbated by the burden of occupational injuries such as LBP and related disabilities (Munabi et al. 2014; Tinubu et al. 2010). It is estimated that in the United Kingdom, each year 12% of nursing personnel will consider a job transfer to reduce their LBP and another 12% – 18% will actually leave the nursing profession because of chronic back pain (Burdorf & Jansen 2006). Work-related musculoskeletal disorders in nursing staff are costly and this includes indirect costs associated with hiring temporary or replacement personnel, overtime to absorb the duties of an injured worker, legal fees, time costs for claim processing, decreased work output following traumatic events and training temporary or replacement personnel (Abuadas 2005).

Nurses are a major part of the workforce in the health sector (Johnson & Emmanuel 2016; Olivier et al. 2009). At a regional hospital in KwaZulu-Natal, the physiotherapy LBP caseload in all nursing categories increased from 15 (30%) to 23 (45%) over three years. About 40% of nurses who required physiotherapy were working in theatre, intensive care units or medical wards. The factors associated with LBP at the hospital remained unclear as to whether the cases are purely occupation related or because of other causes. In addition, some nurses with LBP could have sought treatment from other practitioners outside the hospital. Knowing the factors associated with the high prevalence of LBP will assist in identifying all the nurses at high risk and recommend ways to prevent LBP. For the purpose of this study, current LBP refers to pain that respondents had at the time the study was conducted and lasting for three months or more in an area between the 12th ribs and gluteal fold. The aim of the study was to determine the prevalence and the factors associated with LBP among nurses at a regional hospital in KwaZulu-Natal.

## Methods

### Setting

The study was conducted in a regional hospital in KwaZulu-Natal. The hospital has 900 beds and the services provided include general surgery, obstetrics and gynaecology, medicine, orthopaedics, anaesthesia, intensive care and high care.

### Population

The study population comprised permanently employed nurses working at the regional hospital, either on day shifts or night shifts, between January 2010 and June 2010. The study participants included registered nurses (*n* = 143), enrolled nurses (*n* = 63), nursing assistants (*n* = 31) and matrons (*n* = 4). Registered nurses refer to nurses who completed a four-year training course at a recognised nursing college or university. Enrolled nurses refer to nurses who have completed a two-year training course at a nursing college and nursing assistants are nurses who have completed a one-year training course at a nursing college.

### Sample

The nurses who agreed to participate were screened by research assistants who are qualified physiotherapists to ensure that they meet the inclusion criteria. The list of nurses permanently employed was obtained from the Human Resource Department. Convenience sampling was used; all nurses on duty on the day and night shifts of the day of the visit to a particular unit were approached to participate in the study. There are 450 nurses on duty for day and night shifts on an average day in the hospital. The total number of nurses who were targeted for the study was 300. The expected prevalence of LBP in the population from which the sample was drawn was estimated at 60%. This estimation was based on the number of nurses who reported suffering from LBP during the back care awareness week. A sample size of 282 would provide a 5% precision either side of the population estimate with 95% confidence interval. The desired sample size was increased to 300 to accommodate missing data. All nursing professionals who met the inclusion criteria were eligible to participate in the study.

### Inclusion criteria

All nursing categories permanently employed at the hospital were considered for inclusion in the study.

#### Exclusion criteria

Participants were excluded if they reported to be suffering from LBP as a result of an accident, a deformity, previous spinal injury, pathological backache because of infection, backache because of malignancy, or congenital problems. Student nurses were excluded because they are employed on a temporary employment contract.

## Research design

An exploratory, descriptive study with an analytic, cross-sectional component was implemented.

### Measurement instruments

A standardised self-administered questionnaire was used to determine the prevalence of LBP and its associated risk factors. The questionnaire had been validated and used in a previous study on LBP among therapists (Perreira 2005). The questionnaire was in English as it is the main language of communication in the hospital. The questionnaire was pretested with 10 nurses to ensure that it was user-friendly. Orthopaedic specialists and qualified physiotherapists who work in the spinal and back care field were consulted to establish the face and content validity of the Perreira questionnaire. The variables measured in the questionnaire included personal factors, employment history, LBP history, occupational factors, environmental factors and current LBP history. To ensure that the correct weight and height were recorded, a calibrated weighing scale and a tape measure were used to measure each participant’s actual weight and height. The research assistants assisted the participants to measure their weight and height.

### Data collection

Data were collected from all wards in the hospital. Data collection took place over a period of 1 week (22–29 March 2010). The respondents on day or night shift on the day of data collection in the respective wards completed the questionnaires as guided by the researcher and research assistants.

### Data analysis

The data were captured into Microsoft Excel 2003 and then exported into SPSS 15 for data analysis. Descriptive categorical data were summarised in frequency distribution tables and then analysed for significant associations. The association of risk factors with current LBP was assessed using Pearson’s chi-square test for categorical risk factors and ***t***-test for quantitative risk factors. The lowest risk group was used as the referent group.

### Ethical considerations

All nurses who agreed to participate in the study signed an informed consent form. Questionnaires and signed consent forms were filed separately to ensure anonymity of the questionnaire. Participation was voluntary and participants were informed that they could withdraw from the study without prejudice. The anonymous questionnaires were labelled with unique numbers to ensure confidentiality. Permission to use the questionnaire was obtained from the author.

Ethics approval was granted by the University of KwaZulu-Natal Biomedical Research Ethics Committee (BE161/09), and permission to conduct the study was granted by the KwaZulu-Natal Department of Health (HRKM 013/10) and the hospital management.

## Results

A total of 373 questionnaires were handed out and 242 were completed correctly, yielding a 65% response rate. The period prevalence of reported current LBP was 59% (143/242). The majority of participants were registered nurses (59%), aged between 40 and 49 years (33%) and women (89%) (Table 1). The prevalence of LBP among enrolled nurses (*p* = 0.04) was statistically significant compared with nursing assistants (*p* = 0.19). The prevalence of LBP among participants aged 30–39 years (*p* = 0.03), females (*p* = 0.05) and those who are classified as overweight (*p* = 0.03) was statistically significant. In addition, the prevalence of LBP was high (56%) among those who have been in the nursing profession between 21 and 30 years.

**TABLE 1:** Demographic description of the study sample (*N* = 242).

Characteristics	Category	No.	%
Professional category	Registered nurse	143	59
	Enrolled nurse	63	26
	Nursing assistant	31	13
	Matron	5	2
Age	20–29 years	27	11
	30–39 years	73	30
	40–49 years	80	33
	Older than 50 years	62	26
Sex	Male	27	11
	Female	215	89

The respondents working in obstetrics and gynaecology (49%) reported a higher prevalence of LBP compared with respondents working in orthopaedics and surgery (35%), intensive care units (12%) and medical wards (5%) (Table 2). The association between LBP and working in obstetrics and gynaecology (*p* = 0.01) and orthopaedics and surgery (*p* = 0.002) was statistically significant. Bending and twisting (*p* = 0.002), prolonged sitting (*p* = 0.03), transferring patients (*p* = 0.004) and pushing and pulling (*p* = 0.04) were strongly associated with current LBP (Table 3). The prevalence of LBP was higher among the participants who were on six-month rotations (76%) compared with those on yearly rotation (16%) (Table 4).

**TABLE 2:** Relationship between personal factors and the current back pain.

Personal	Category	Low back pain *n* (%)	95% CI	*p*
Professional category	Registered nurse	40 (17)	referent	-
	Enrolled nurse	120 (50)	0.9–3.4	0.04
	Nursing assistant	82 (33)	0.8–3.9	0.19
Age	20–29	35 (15)	referent	-
	30–39	112 (46)	1.2–7.8	0.03
	40–49	25 (10)	0.7–4.6	0.24
	Above 50	70 (29)	0.9–6.2	0.09
Gender	Male	94 (39)	referent	-
	Female	148 (61)	1.1–7.2	0.05
BMI	Underweight	35 (14)	referent	-
	Overweight	140 (58)	1.2–5.4	0.03
	Obese	67 (28)	1.5–5.8	0.08
Ward	Medical	11 (5)	referent	-
	Intensive Care Unit	28 (11)	1.2–4.2	0.29
	Obstecrics /Gynaecology	118 (49)	1.4–7.9	0.01
	Ortho/surgery	85 (35)	1.9–11.2	0.002
Years in nursing	0–10	30 (12)	-	-
	11–20	56 (23)	0.46–1.55	0.84
	21–30	136 (56)	0.52–2.07	1.03
	31–40	20 (9)	0.44–4.19	1.78
Years at hospital	0–10	55 (23)	-	-
	11–20	78 (32)	0.46–1.51	0.83
	21–30	83 (34)	0.39–1.74	0.83
	31–40	26 (11)	0.22–1.17	1.27

CI, confidence interval.

**TABLE 3:** Occupational and presence of current low back pain.

Occupational factors	Low back pain *n* (%)	95% CI	*p*
Working in awkward position	15 (6)	referent	-
Bending or twisting	194 (80)	1.4–5.3	0.002
Lifting	179 (74)	0.8–4.2	0.17
Prolonged position	186 (77)	1.1–4.1	0.03
Repetitive task	58 (24)	0.7–2.9	0.28
Responding to sudden movement	36 (15)	0.7–6.7	0.18
Transferring patients	46 (19)	1.4–5.2	0.004
Working in cramped position	22 (9)	0.9–4.8	0.09
Pushing or pulling	46 (19)	1.1–3.7	0.04

CI, confidence interval.

**TABLE 4:** Environmental factors and presence of current low back pain.

Environmental factors	Category	Low back pain *n* (%)	CI 95%	*p*
Rotation	Day shift	123 (51)	-	-
	Night shift	119 (49)	0.34–1.51	0.37
	3-month rotation	20 (8)	-	-
	6-month rotation	183 (76)	1.35–18.47	0.02*
	Yearly rotation	39 (16)	0.44–3.26	0.72
Assistance with handling	Yes	129 (53)	-	-
	No	113 (47)	0.23–2.71	0.33
Assistive devices with patient handling	Yes	47 (9)	-	-
	No	195 (81)	0.21–2.13	0.26
Adjustable surfaces	Yes	33 (14)	-	-
	No	209 (86)	0.50–1.89	0.23
Work surface height	Too high	55 (23)	-	0.12
	Too low	88 (36)	0.16–1.75	0.55
	Neither low nor high	99 (41)	0.21–1.96	0.64
Chair height	Too high	40 (17)	-	-
	Too low	20 (8)	0.42–1.67	0.54
	Neither low nor high	182 (75)	0.32–1.78	0.34

## Discussion

The period prevalence of current LBP among nurses in this study was 59%. This is comparable with the findings of a study by Olivier et al. (2009) among 354 hospital employees in a district hospital in South Africa, where nursing staff were found to have a higher prevalence (59%) of LBP compared with other health professionals, such as doctors and allied health professionals (Olivier et al. 2009). The high prevalence of LBP among nurses in the district hospital was attributed to the manual handling of patients, a physical load that is carried out primarily by nurses (Olivier et al. 2009). The factors associated with the high prevalence in this study included prolonged position, working in cramped position, bending or twisting, and pushing or pulling.

In this study, a higher percentage of enrolled nurses (50%) experienced current LBP compared with nursing assistants (33%) and professional nurses (17%). The findings of this study concur with the results of a study carried out in Britain reporting that professional nurses had a lower prevalence of back pain compared with the enrolled nurses and nursing assistants (Hollingdale 1997). The lower prevalence of back pain among professional nurses in the British study was attributed to the fact that professional nurses are less involved in the manual handling of patients because of their organisational and management responsibilities (Hollingdale 1997). Enrolled nurses’ and nursing assistants’ jobs involve more direct patient care, such as moving and transferring patients, and transporting material and medical devices (Hollingdale 1997). This is similar to enrolled nurses’ and nursing assistants’ South African context. In addition, a study in Greece that compared LBP in relation to nurses’ education suggested that the high prevalence among enrolled nurses and nursing assistants was because of the shorter duration of training, not giving enough time to cover prevention issues related to musculoskeletal injuries and manual handling of patients during training (Zoe 2008).

In comparison, a higher proportion (61%) of women suffered from current LBP than men (37%). These findings are comparable with the results of a Nigerian study that reported a 68% prevalence for female nurses and 32% for male nurses (Sikiru & Hanifa 2010). A study in Yemen concluded that female nurses were more at risk of developing LBP (Ghilan et al. 2013). The difference in prevalence may be related to the anatomical, physiological and structural differences between women and men (Sikiru & Hanifa 2010). In addition, in the African context, women tend to do more household chores compared to men; thus, LBP may be a reflection of the accumulation of a number of exposures at work and at home.

The prevalence of current LBP was higher among respondents who were overweight (58%). A positive, statistically-significant association was found between being overweight and current LBP. The effect of high body mass index (BMI) on LBP has been reported in some studies (Andrusaitis, Oliveira & Barros Filho 2006; Heuch et al. 2010; Shiri et al. 2010). In contrast, other studies have reported no links between obesity and LBP (Ibrahimi-Kaçuri et al. 2015; Mirtz & Greene 2005).

A high proportion of nurses working in obstetrics and gynaecology, and orthopaedics and surgery were reported to be experiencing current LBP. The results of this study correspond with the findings of previous studies, concluding that nurses working in surgery, and obstetrics and gynaecology departments, have the highest prevalence of LBP (Sikiru & Hanifa 2010; Tezel 2005). The prevalence of LBP was higher among nurses who have been in the profession for over 20 years, which could suggest that increased occupational exposure contributes to the development of LBP among nurses.

Lifting, bending, pulling, pushing and sustained position were identified as risk factors for current LBP in this study, and several associations were found to be statistically significant. Respondents in a study in a Nigerian hospital associated LBP with heavy physical work, sustained position and lifting (Tinubu et al. 2010). The findings were also confirmed by a study that was conducted among Japanese nurses. In the Japanese study, the risk of developing LBP for nurses involved in manual handling was high, with an odds ratio of 16.7, when compared with nurses who were not involved in manual handling of patients (Smith et al. 2003). Further research needs to be conducted to investigate the influence of cumulative effects of various manual handling activities on LBP. There was a strong association between respondents who were on six-month rotations and the presence of chronic LBP which was statistically significant (*p* = 0.04). Job rotation in the nursing profession has been reported to be associated with stress and low job satisfaction (Ho et al. 2009; Olivier et al. 2009). Stress and low job satisfaction has been associated with LBP.

### Limitations

This study used a cross-sectional study design and it is difficult to establish causality because the time sequence is not clear. Healthy worker selection could have biased the results because of the fact that those suffering from LBP might have left the nursing profession or changed to other jobs, prior to data collection. The respondents were unable to state about time spent doing activities like lifting, transfers, bending, sitting and standing. In order to draw accurate conclusions regarding these activities, this should be assessed by direct participant observation. The questionnaire did not include recreational activities and LBP might have been aggravated or ameliorated by other activities such as exercise and household chores.

It was not possible in this study to explain the differences in prevalence in these wards because of limited information. Further research accounting for nursing category, characteristics of patients and ergonomic task analysis is needed to explain the differences in the LBP prevalence among nurses working in these wards. Because of the small sample size, this study could not stratify by nursing category to ascertain whether nursing assistants reported occupational factors differently from the other groups.

## Conclusions

Low back pain is an occupational hazard among nurses. Occupational factors are strongly associated with LBP. Education programmes on prevention and workplace interventions are required in order to reduce occupational injuries.

## Further research and recommendations

More research is needed to explore the relationship between nursing category and the prevalence of LBP in the South African context. A participant observation study evaluating application of skills is required. Furthermore, an enquiry on nurses’ perceptions of the possible causes of LBP and their practices is useful. The hospital should be well equipped with appropriate assistive devices for manual handling of patients. Induction courses for back care for nurses must be conducted for all new recruits. Regular in-service training on back care and ergonomics must be conducted in various wards to assist nurses to refresh manual patient handling techniques. Nurses should be taught on the importance of weight management and physical fitness in preventing LBP.
